# Supplementary figures and images for: Feasibility of two screen media reduction interventions: Results from the SCREENS pilot trial
Source: PLoS One. 2021 Nov 15;16(11):e0259657. doi: 10.1371/journal.pone.0259657 (PMC8592478; doi:10.1371/journal.pone.0259657)

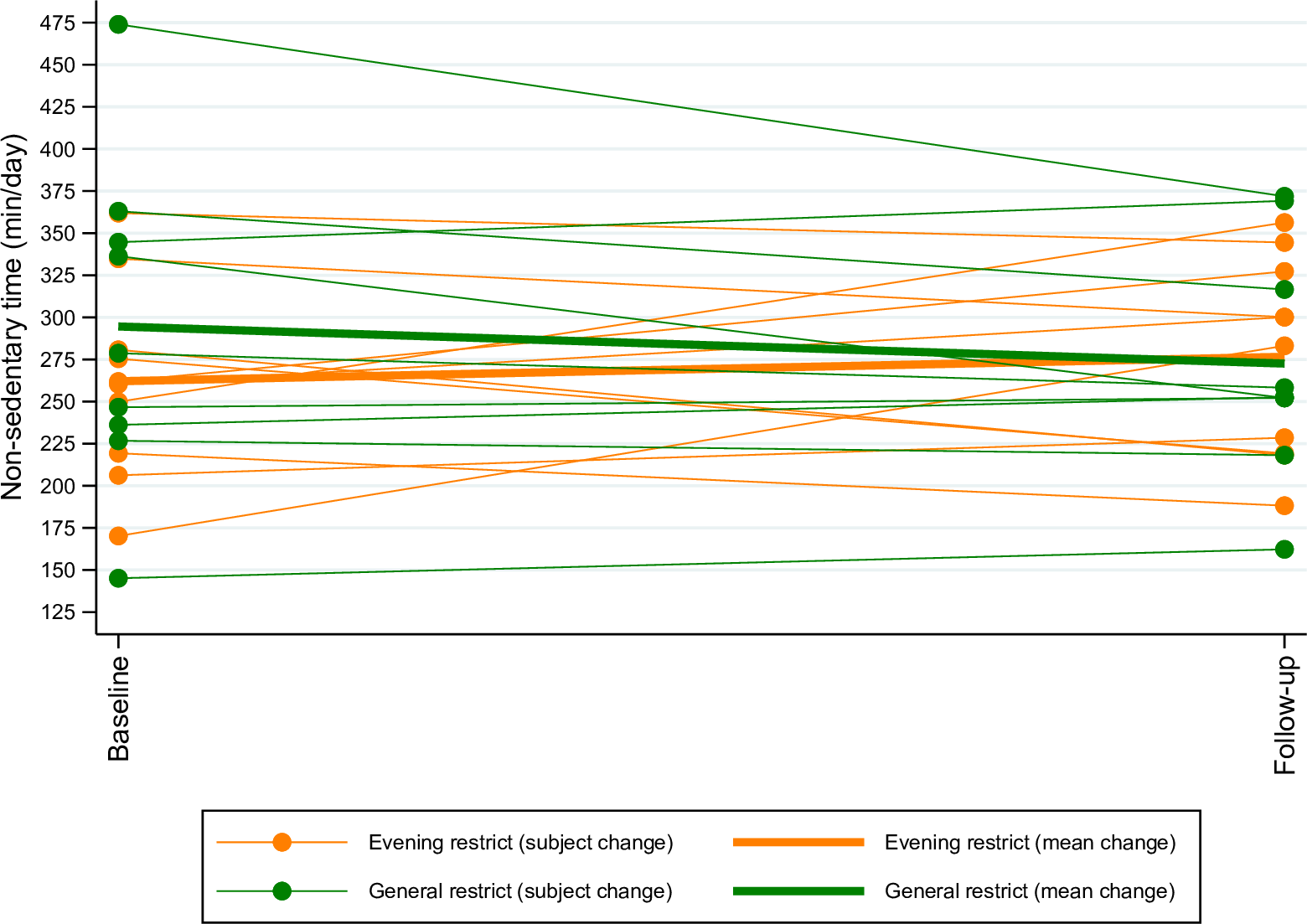

Supplement: S1 Fig — The figure above illustrates the change in non-sedentary time (min/day) for adults participating in the SCREENS pilot trial. The data is parsed based on group allocation (Evening Restrict group: orange, General Restrict group: green). A thick best fit line for each group has been added to display direction of change in mean non-sedentary time from baseline to follow-up, in each group. (TIF) [file pone.0259657.s003.tif]
