# Supplementary material for: Feasibility of two screen media reduction interventions: Results from the SCREENS pilot trial
Source: PLoS One. 2021 Nov 15;16(11):e0259657. doi: 10.1371/journal.pone.0259657 (PMC8592478; doi:10.1371/journal.pone.0259657)
Supplement: S1 Table — (DOCX) [file pone.0259657.s004.docx]

Supplementary Table 1. Characteristics of participants from the source population, survey non-respondents, survey respondents who were ineligible or eligible to participate in the feasibility trial based on initial criteria, and feasibility trial participants.

|  | Feasibility trial participants | Survey respondents eligible to participate in the trial based on at least initial criteria but who did not participate in feasibility trial* | Survey respondents ineligible to participate in the trial based on initial or secondary criteria** | Non-respondents | Source population |
| --- | --- | --- | --- | --- | --- |
| N (parent and child dyads) | 12 | 90 | 292 | 1,292 | 1,675 |
| Age of parent, yrs | 42 (39-46) | 41.0 (38.0-45.0) | 40.0 (36.5-44) | 40.0 (36.0-43.0) | 40.0 (36.0-43.0) |
| Age of child, yrs | 9 (7-10) | 9.0 (7.0-10.0) | 8.0 (7.0-9.0) | 8.0 (7.0-9.0) | 8.0 (7.0-9.0) |
| Gender of parent, % female | 57.9 | 63.3 | 68.2 | 59.0 | 60.8 |
| Gender of child, % female | 28.6 | 36.7 | 45.6 | 51.0 | 49.1 |
| Educational attainment of parent – ISCED, % (≤2, 3-5, ≥6) | 5.3/42.1/52.6 | 2.2/43.3/54.4 | 7.1/41.0/51.9 | - | - |
| Children’s total recreational screen media use on weekdays | 2.9 (1.9-3.6) *** | 2.5 (1.8-3.3) | 1.5 (0.8-2.8) | - | - |
| Children’s total recreational screen media use on weekend days | 4.6 (3.3-5.1) *** | 4 (2.5-5.5) | 2.5 (1.5-4.0) | - | - |

*Initial eligibility criteria based on questionnaire responses were: adults’ total screen media above the median for all respondents, no children younger than 4 years of age residing in the household, adults had to work daytime hours (between 6 AM and 6 PM), the responding adult had to reside full time in the household with the child (parent and child dyad).

**Additional secondary eligibility criteria were assessed via telephone (see methods section).

***Only based on information from the parent-child dyad (dyads were randomly selected among members of the household).
